# Klotho Endows Hepatoma Cells with Resistance to Anoikis via VEGFR2/PAK1 Activation in Hepatocellular Carcinoma

**DOI:** 10.1371/journal.pone.0058413

**Published:** 2013-03-13

**Authors:** Lin Chen, Haiou Liu, Jing Liu, Yu Zhu, Le Xu, Hongyong He, Heng Zhang, Shanshan Wang, Qian Wu, Weisi Liu, Yidong Liu, Deng Pan, Shifang Ren, Jiejie Xu, Jianxin Gu

**Affiliations:** 1 Department of Biochemistry and Molecular Biology, School of Basic Medical Sciences, Fudan University, Shanghai, China; 2 Department of Urology, Zhongshan Hospital, Shanghai Medical College, Fudan University, Shanghai, China; 3 Department of General Surgery, Zhongshan Hospital, Shanghai Medical College, Fudan University, Shanghai, China; 4 School of Medicine, Nantong University, Nantong, China; University of Kentucky College of Medicine, United States of America

## Abstract

Klotho was originally characterized as an aging suppressor gene that predisposed Klotho-deficient mice to premature aging-like syndrome. Although Klotho was recently reported to exhibit tumor suppressive properties during various malignant transformations, the functional role and molecular mechanism of Klotho in hepatocarcinogenesis remains poorly understood. In our present study, immunohistochemical Klotho staining levels in a clinical follow-up of 52 hepatoma patients were significantly associated with liver cirrhosis, tumor multiplicity and venous invasion. The overall survival rate of hepatoma patients with high Klotho expression was significantly lower than those patients with low Klotho expression. Moreover, Klotho overexpression increased cellular migration, anchorage-independent growth, and anoikis resistance in hepatoma cells. Klotho overexpression elevated p21-activated kinase 1 (PAK1) expression and shRNA-mediated PAK1 knockdown and kinase activity inhibition with kinase dead mutant PAK1 K299R coexpression or allosteric inhibitor IPA3 treatment reversed anoikis resistance in Klotho-overexpressed hepatoma cells. More importantly, the pivotal significance of upregulated VEGFR2 protein levels mediated by Klotho expression was confirmed by VEGFR2 inhibitor Axitinib and blocking antibody treatment in hepatoma cells. Axitinib treatment sensitized anoikis was reversed by constitutive active mutant PAK1 T423E coexpression in Klotho-overexpressed hepatoma cells. Conversely, knockdown of Klotho reduced VEGFR2/PAK1 dependent anoikis resistance, which could be reversed by PAK1 T423E. These results revealed a novel oncogenic function of Klotho in promoting anoikis resistance via activating VEGFR2/PAK1 signaling, thus facilitating tumor migration and invasion during hepatoma progression, which could provide a putative molecular mechanism for tumor metastasis.

## Introduction

Primary liver cancer, which consists predominantly of hepatocellular carcinoma (HCC), ranks as the fifth most prevalent malignancy and the third leading cause of cancer mortality worldwide [1]. Despite advances in many aspects of HCC treatment, including liver transplantation, surgical resection, and locoregional therapies [2], recurrence or metastasis is quite common in patients who have had a resection and survival rate is 30% to 40% at 5 years postoperatively [3]. Thus, molecular mechanisms underlying hepatoma metastasis is urgently needed to determine for developing potential molecular targeting therapeutic options in HCC patients with metastasis.

Metastasis requires that tumor cells detach from the primary matrix or cell-cell anchors that control tissue architecture [4]. Under normal circumstances, epithelial cells undergo anoikis, a specialized form of apoptosis, which occurs on cells due to inadequate or inappropriate cell-matrix interactions through disruption of anchorage-dependent cell growth [5]. Metastatic tumor cells therefore must be resistant to anoikis to survive during dissemination and colonisation of ectopic sites [4]. The importance of anoikis resistance in liver cancer metastasis was elegantly shown in our previous studies where p21-activated kinase 1 (PAK1) has been identified as a key mediator for hepatoma resistance to anoikis [6], [7]. However, the initial upstream stimulator for PAK1 activation in hepatocarcinogenesis remains obscure and addresses our research interest herein.

Klotho is recently identified as a senescence-suppressing gene [8]. Deficiency of Klotho in mice causes a syndrome resembling human ageing including arteriosclerosis, osteoporosis, endothelial dysfunction, Parkinsonian gait and cognitive impairment [8], [9], [10], [11]. In humans, Klotho polymorphisms have been associated with both reduced human longevity and coronary-artery disease [12]. Alternative splicing at the RNA level of the Klotho gene results in two transcripts in which one encodes a single-pass membrane form of the protein, whereas the other transcript encodes a putative secreted form that acts as a humoral factor [13]. Although Klotho has been characterized as a potential tumor suppressor in tumorigenesis of various human cancers, the functional role and molecular mechanism of Klotho in hepatocarcinogenesis remains poorly understood.

In the present study, we found for the first time that high immunohistochemical Klotho staining levels were significantly associated with liver cirrhosis, tumor multiplicity, venous invasion, and poor overall survival in a clinical follow-up of 52 hepatoma patients. Furthermore, Klotho expression conferred hepatoma cells with resistance to anoikis via activating VEGFR2/PAK1 signaling, which could be reversed by inhibition with PAK1 allosteric inhibitor IPA3 or VEGFR2 inhibitor Axitinib administration in Klotho-overexpressed hepatoma cells. These results identified Klotho/VEGFR2/PAK1 as a crucial molecular pathway underlying resistance to anoikis for hepatoma cells, which could open a new avenue for molecular targeting therapeutic intervention with anoikis resistance in HCC patients with metastasis.

## Materials and Methods

### Ethics Statement

Ethical approval was granted by the Ethics Committee of the Fudan University. Written informed consent was obtained for the acquisition and use of patient tissue samples and anonymized clinical data.

### Cell lines and patient samples

One immortalized liver cell line (L02) and five hepatoma cell lines (HepG2, Huh7, BEL7402, BEL7404, and PLC/PRF/5) were cultured in DMEM supplemented with 10% FBS in 5% CO_2_ incubator at 37°C. Fifty-two pairs of human hepatoma and peripheral nontumor tissues after surgical resection were collected from HCC patients in Nantong Tumor Hospital (Nantong University, Jiangsu, China) from 2003 to 2005. Use of human tissues with informed consent was approved by the Institutional Review Board of Fudan University.

### Construction of plasmids

Expression plasmid encoding Klotho was kindly provided by Dr. Ci-Di Chen (Boston University School of Medicine, Boston, MA). The plasmids containing wild type PAK1, kinase dead mutant PAK1 K299R, and constitutive active mutant PAK1 T423E were constructed as described previously [14]. All plasmid constructs were confirmed by DNA sequencing.

### Plasmid transfection and RNA interference (RNAi)

Transient and stable transfections with various plasmids were performed as previously described [15]. To judge the efficiency of transfection, cells were cotransfected with a GFP-expressing plasmid (pEGFP-N1) at a ratio of 10∶1 (target gene: GFP plasmid). Efficiency of transient transfection under the conditions of these experiments was judged by western blot analysis. The shRNA against PAK1 gene PAK1 shRNA (h),against Klotho gene Klotho shRNA (h) and corresponding control shRNA (Santa cruz Biotechnology) were used for RNA interference as described previously [6]. Gene silencing effect was confirmed by Western blot analysis and RT-PCR at 48 hours posttransfection.

### Immunohistochemistry staining and evaluation

A total of 52 pairs of HCC samples were H&E stained and immunohistochemically analyzed as described previously [16]. Primary anti-Klotho antibody (Abcam, Cambridge, MA) and anti-PAK1 antibody (Santa cruz Biotechnology) were used for immunohistochemistry analysis and the intensity of immunohistochemistry staining in the tumor tissues was scored independently by two pathologists according to the intensity and percentage of positive cells as described previously [17], [18]. The immunostaining levels were scored as 0 (negative), 1+ (weakly positive), 2+ (moderately positive), or 3+ (strongly positive). High expression in tumor cells was defined as score ≥2+.

### Western blot, cell migration assay, colony formation assay and immunofluorescence staining

Protein extraction from cultured cells and Western blot analysis were carried out as previously described [15]. Primary antibodies used in Western blot included those against Klotho (Abcam, Cambridge, MA), GFP,PAK1 and GAPDH (Santa Cruz Biotechnology, Santa Cruz, CA). Cell migration,colony formation assay and immunofluorescence staining were performed as described previously [15], [19].

### *In vitro* anoikis assay, Caspase 3/7 activity assay, Annexin V/PI staining, and TUNEL assay

*In vitro* anoikis assay was performed with cell culture on poly-HEMA-coated 6-well tissue culture plates for 48 hours as described previously [6]. Caspase 3/7 activity assay, Annexin V/PI staining, and TUNEL assay were performed as described previously [6].

### Flow cytometry

Flow cytometry analysis for VEGFR2 expression on hepatoma cells was performed with anti-VEGFR2-Percp (R&D Systerms, Minneapolis, MN) as described previously [20], [21]. All flow cytometry data were acquired on a BD FACSCalibur (BD Biosciences) and analyzed by FlowJo software (Tree Star, San Carlos, CA).

### Statistical Analysis

Experimental data were presented as mean±SEM of at least three independent replicates through analyzing with GraphPad Prism 5 (GraphPad Software, La Jolla, CA) and assessing comparisons between different groups by the Student's *t* test. The correlation between Klotho expression and clinicopathological features was assessed using the *t* test or Fisher's exact test with SPSS version 17.0 statistical software package (SPSS Inc., Chicago, IL). Survival was calculated from the date of surgery to date of death or last follow-up. Survival curves were estimated using the Kaplan-Meier method, and log-rank test was used to compute differences between the curves. Differences were considered significant at values of *P*<0.05.

## Results

### Klotho expression correlates with clinicopathologic features and overall survival of HCC samples

**Figure 1:**
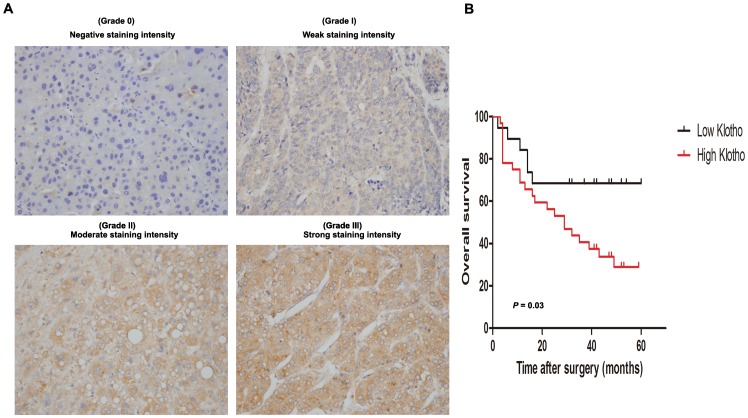
Immunohistochemical Klotho staining in tumor tissues associates with overall survival of hepatoma patients. (A) Representative photomicrographs of immunohistochemical Klotho staining in human HCC tissues: Grade 0 for negative staining intensity, Grade I for weak staining intensity, Grade II for moderate staining intensity, and Grade III for strong staining intensity (original magnification, ×400). (B) Kaplan-Meier survival analysis between HCC patients with high Klotho expression (n = 33) and those with low Klotho expression (n = 19). *P* value was calculated by log-rank test.

To investigate the clinical significance of Klotho in hepatocarcinogenesis, we first evaluated immunohistochemical Klotho staining intensity in tumor specimens from 52 patients with HCC and classified these hepatoma samples into four categories: negative staining intensity (grade 0) for 1 case, weak staining intensity (grade I) for 10 cases, moderate staining intensity (grade II) for 18 cases, and strong staining intensity (grade III) for 23 cases (Figure 1A). Taken the percentage of positive cancer cells on each slide ranging from 10% to 90% into account, the cutoff value of Klotho staining was set on the median H-score at 1.5 and 52 HCC patients were divided into high Klotho expression group (H-score>1.5) with 33 (63.46%) cases and low Klotho expression group (H-score≤1.5) with 19 (36.54%) cases (Table 1).

**Table 1:** Correlation between Klotho expression and patient characteristics.

Characteristic	Patients (n = 52)		Klotho expression		*P* value*
	Number	%	High (n = 33)	Low (n = 19)	
Age (years)†	52	100.00	53.21±7.62	55.52±8.74	NS
Gender					
Male	46	88.46	28	18	
Female	6	11.54	5	1	NS
HBsAg					
Positive	27	51.92	20	7	
Negative	25	48.08	13	12	NS
Liver cirrhosis					
No	30	57.69	15	15	
Yes	22	42.31	18	4	0.023
Tumor size (cm)†	52	100.00	7.61±1.10	6.92±1.09	NS
Tumor multiplicity†	52	100.00	1.3±0.11	1.11±0.01	0.007
Microsatellite nodules					
Absent	40	76.92	23	17	
Present	12	23.08	10	2	NS
Tumor encapsulation					
Absent	13	25.00	9	4	
Present	39	75.00	24	15	NS
Intrahepatic metastasis					
Absent	46	88.46	27	19	
Present	6	11.54	6	0	NS
Venous invasion					
Absent	44	84.62	25	19	
Present	8	15.38	8	0	0.021
Tumor stage					
I–II	41	78.85	26	15	
III–IV	11	21.15	7	4	NS
BCLC stage					
0	4	7.69	2	2	
A	15	28.85	8	7	
B	23	44.23	16	7	
C	9	17.31	6	3	
D	1	1.92	1	0	NS

* *P*<0.05 is considered statistically significant, NS means statistically no significant (*t* test for continuous variables and fisher's exact test for categorical variables).

† Results of continuous variables are expressed as mean±standard deviation.

Immunohistochemical staining of Klotho levels were statistically analyzed to determine their relationship with clinicopathologic features of the 52 liver cancer patients. As shown in Table 1, Klotho overexpression strongly correlated with liver cirrhosis (*P* = 0.023), tumor multiplicity (*P* = 0.007), and venous invasion (*P* = 0.021) of HCC patients, whereas it was not associated with age, gender, HBsAg, tumor size, microsatellite nodules, tumor encapsulation, intrahepatic metastasis,tumor stage, or BCLC stage. Moreover, Kaplan-Meier survival analysis indicated that the overall survival rate of hepatoma patients with high Klotho expression was significantly lower than those with low Klotho expression (*P* = 0.03) (Figure 1B). These data suggest the potential contribution of Klotho expression in HCC progression.

### Klotho expression promotes cellular migration, anchorage-independent growth, and anoikis resistance of hepatoma cells

**Figure 2:**
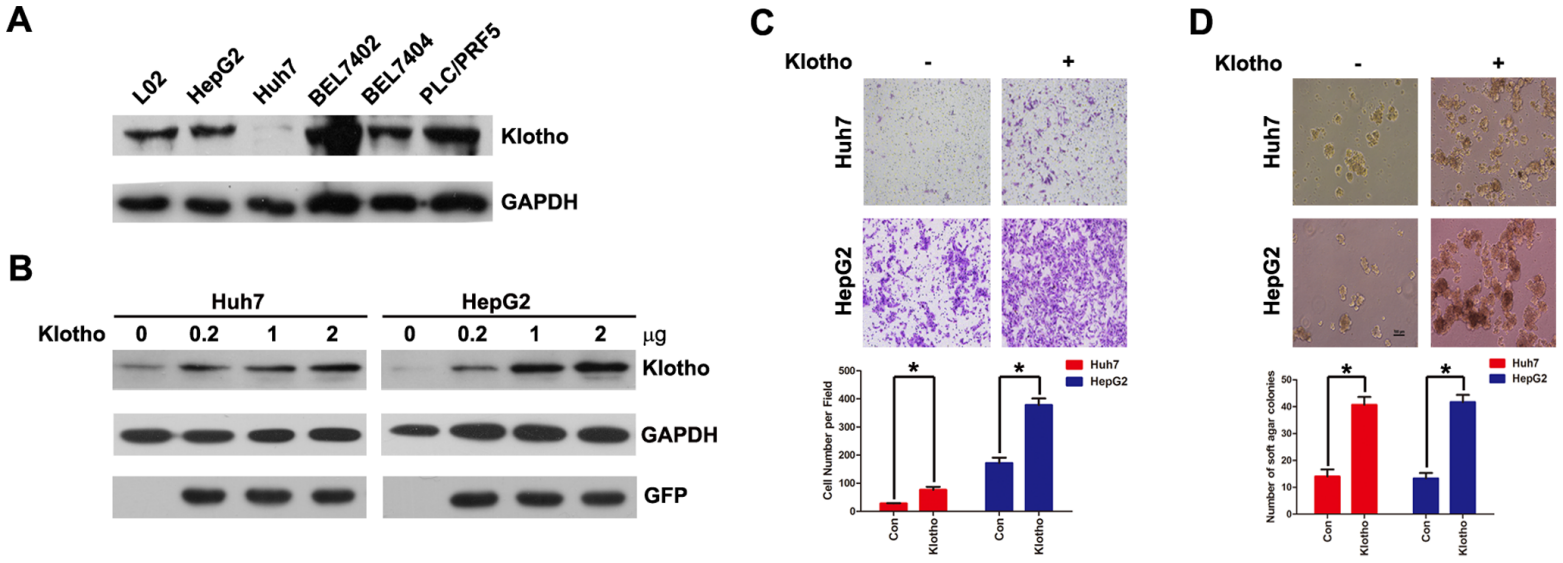
Klotho expression increases hepatoma cell migration and anchorage-independent growth. (A) Western blot analysis of Klotho and GAPDH protein levels in L02, HepG2, Huh7, BEL7402, BEL7404, and PLC/PRF/5 cells. (B) Western blot analysis of Klotho,GFP and GAPDH protein levels in Huh7 and HepG2 cells transiently transfected with empty vector and increased concentration of Klotho plasmid, respectively. (C) Cell migration assay and (D) Colony formation assay in Huh7 and HepG2 cells transiently transfected with empty vector and Klotho plasmid, respectively. The graphic profiles represent the mean of three independent replicates in each group with standard error bars and were statistically analyzed with a *t* test (*, *P*<0.05).

To address the potential function of Klotho expression in HCC progression, we selected two hepatoma cell lines (Huh7 and HepG2) with low Klotho expression to explore functional alterations after Klotho overexpression (Figure 2A, B). Cell migration assay demonstrated that ectopic expression of Klotho increased hepatoma cell migration (Figure 2C). Moreover, colony formation assay revealed that Klotho expression promoted significantly anchorage-independent growth of hepatoma cells (Figure 2D).

**Figure 3:**
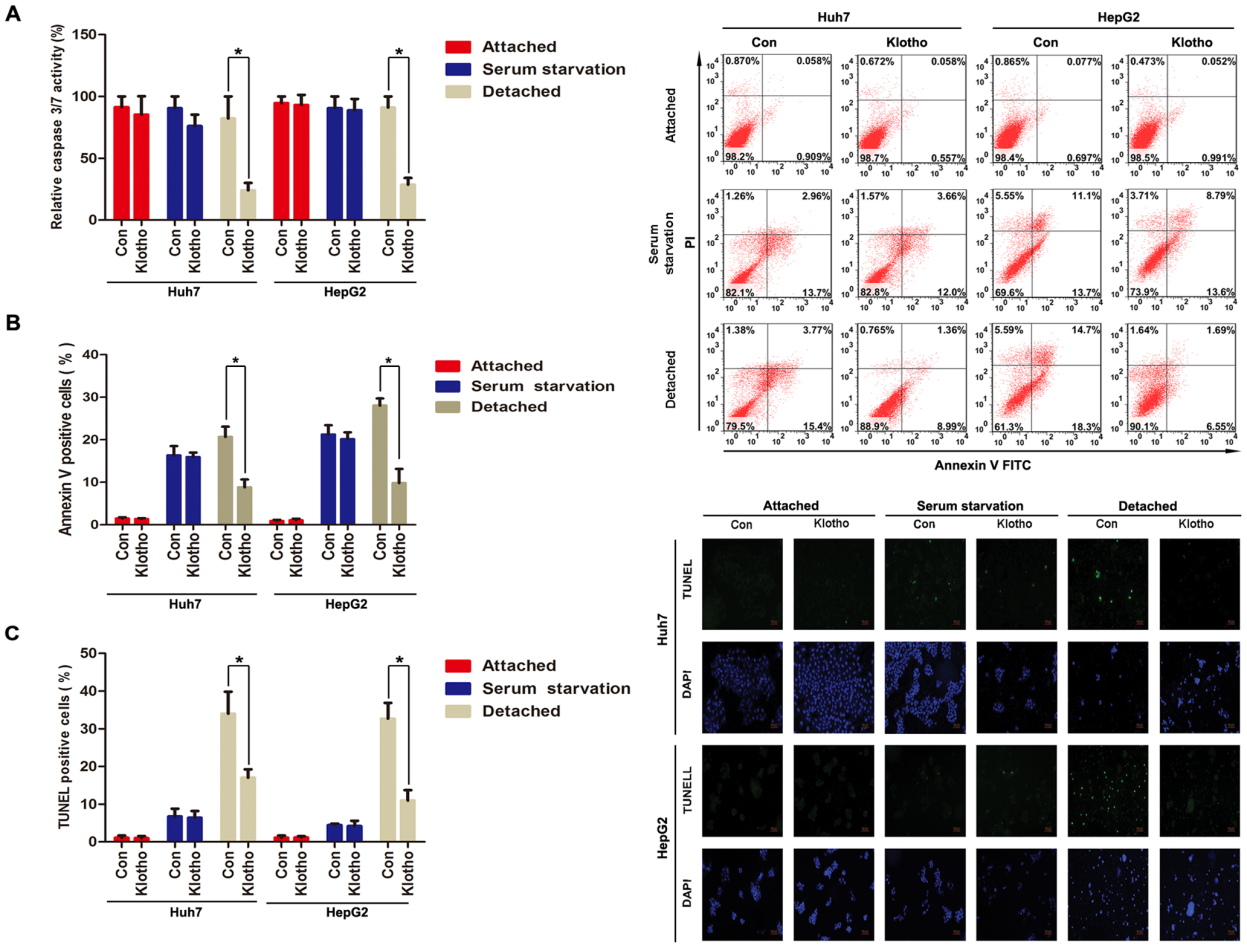
Klotho expression endues hepatoma cells with resistance to anoikis. (A) Caspase 3/7 activity assay, (B) Annexin V/PI staining, and (C) TUNEL assay for Huh7 and HepG2 cells transiently transfected with empty vector and Klotho plasmid, respectively, during attached, serum starvation, and detached status for 48 hours. The graphic profiles represent the mean of three independent replicates in each group with standard error bars and were statistically analyzed with a *t* test (*, *P*<0.05).

Since anoikis resistance is prerequisite for acquisition of anchorage-independent growth in malignant cells [22], we propose that anchorage-independent growth obtained from Klotho expression might be attributable to Klotho-induced anoikis resistance. To validate this hypothesis, *in vitro* anoikis assay for Huh7 and HepG2 cells was performed in the presence or absence of Klotho overexpression. Elevated caspase 3/7 activities after detachment in these two hepatoma cells were significantly rescued by Klotho expression (Figure 3A). Consistent with this phenomenon, increased apoptosis after detachment in Huh7 and HepG2 cells was alleviated significantly by Klotho expression using Annexin V/PI staining (Figure 3B) and TUNEL assay (Figure 3C). These results indicate that anchorage-independent growth obtained after Klotho expression is attributed partly to Klotho-induced anoikis resistance.

**Figure 4:**
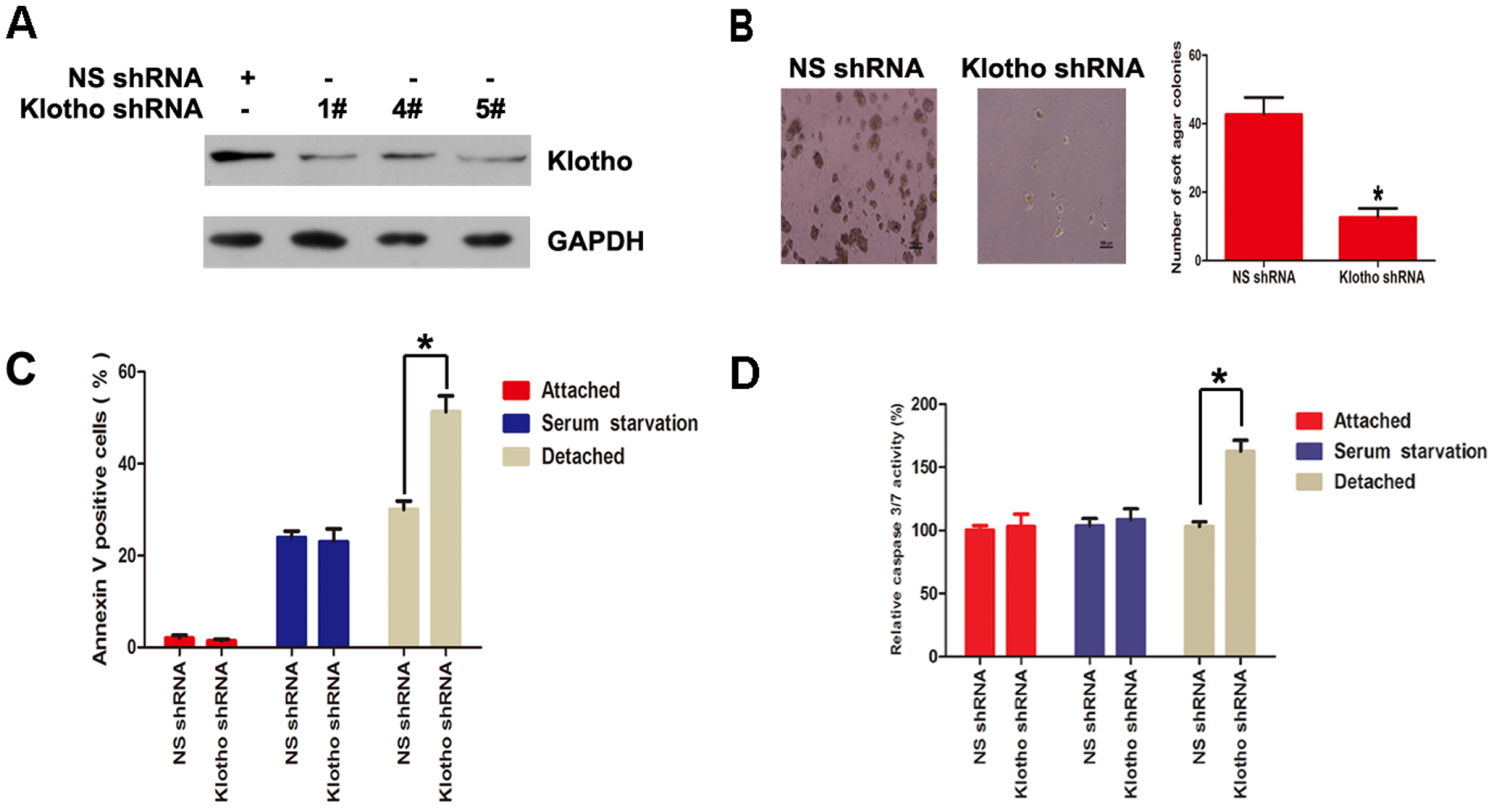
Knockdown Klotho attenuates anoikis resistance of hepatoma cells. (A) Western blot analysis of Klotho and GAPDH protein levels in BEL7402 cells stably transfected with nonspecific shRNA, Klotho 1# shRNA, Klotho 4# shRNA, or Klotho 5# shRNA, respectively. (B) Colony formation assay, (C) Annexin V/PI staining, and (D) Caspase 3/7 activity assay for BEL7402 cells stably transfected with nonspecific shRNA or Klotho 1# shRNA, respectively, during attached, serum starvation, and detached status for 48 hours. The graphic profiles represent the mean of three independent replicates in each group with standard error bars and were statistically analyzed with a t test (*, *P*<0.05).

To further elucidate the pivotal role of Klotho in anchorage-independent growth and anoikis resistance, Klotho was knockdown by shRNA in BEL7402 cells, and 1# shRNA was chosen for further investigation (Figure 4A). Colony formation assay showed that Klotho knockdown decreased anchorage-independent growth (Figure 4B). Furthermore, Annexin V/PI staining (Figure 4C) and Caspase 3/7 activity assay (Figure 4D) showed that anoikis is increased after detachment in BEL7402 cells with Klotho knockdown.

### Klotho expression confers resistance of hepatoma cells to anoikis via PAK1 activation

**Figure 5:**
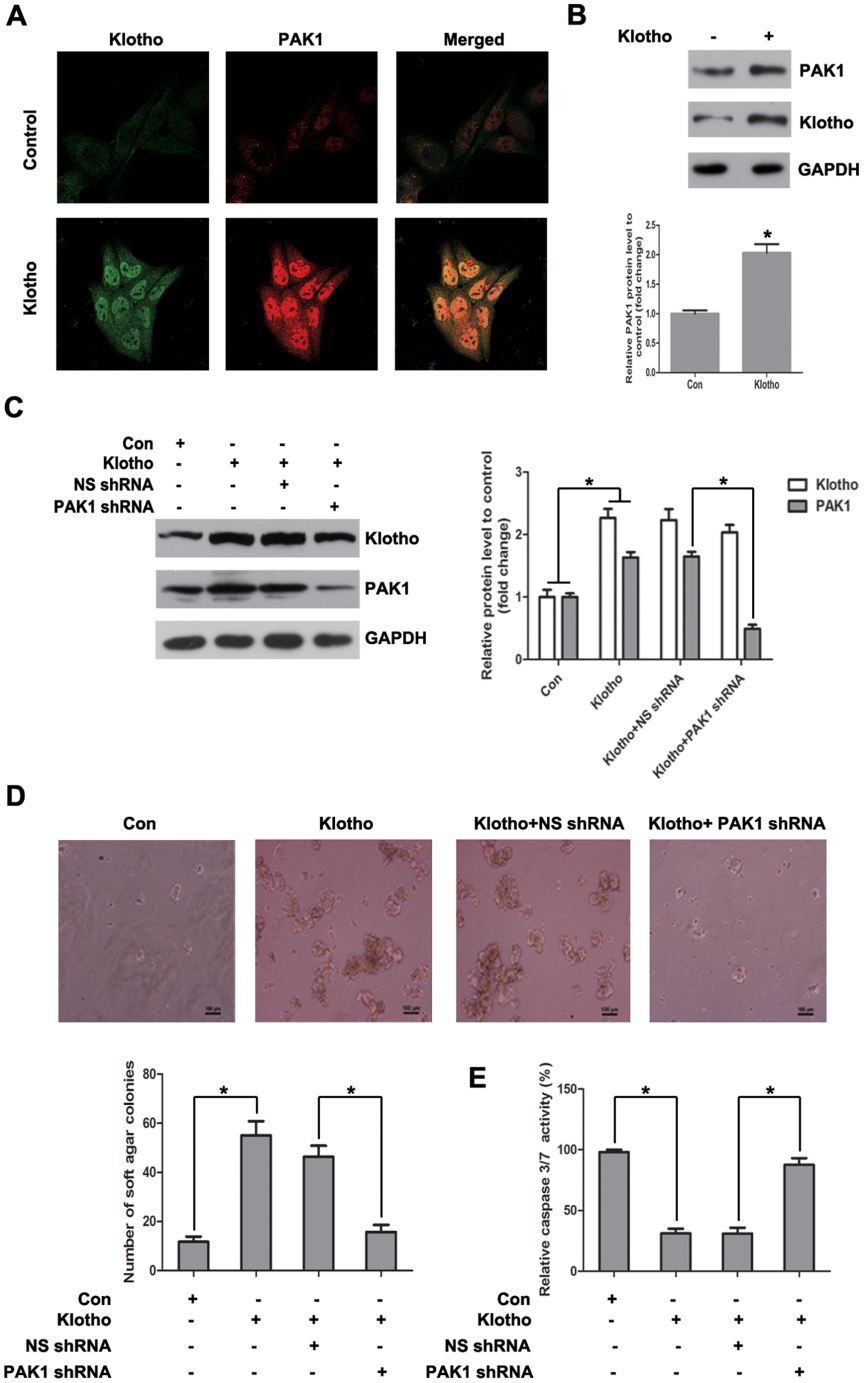
PAK1 upregulation is involved in Klotho-induced anoikis resistance. (A) Immunofluorescence staining of PAK1 and Klotho, and (B) Western blot analysis of PAK1, Klotho and GAPDH protein levels for Huh7 cells transiently transfected with empty vector and Klotho plasmid, respectively. (C) Western blot analysis of PAK1, Klotho and GAPDH protein levels for Huh7 cells stably transfected with empty vector and Klotho plasmid, respectively, without or with nonspecific shRNA or PAK1 shRNA cotransfection together. (D) Colony formation assay and (E) Caspase 3/7 activity assay after 48 hours detachment for Huh7 cells stably transfected with empty vector and Klotho plasmid, respectively, without or with nonspecific shRNA or PAK1 shRNA cotransfection together. The graphic profiles represent the mean of three independent replicates in each group with standard error bars and were statistically analyzed with a *t* test (*, *P*<0.05).

In our previous studies, we have identified PAK1 activation is crucial for resistance of hepatoma cells to anoikis [6], [7]. Klotho expression was found to upregulate PAK1 protein level in Huh7 cells by immunofluoresence staining (Figure 5A) and western blot (Figure 5B). In order to elucidate the role of PAK1 in Klotho-mediated anoikis resistance, PAK1 was knockdown by PAK1 shRNA in Klotho-overexpressed Huh7 cells (Figure 5C). Colony formation assay showed that increased anchorage-independent growth of Klotho-overexpressed Huh7 cells was blunted by shRNA-mediated PAK1 knockdown (Figure 5D). In addition, Caspase 3/7 activity assay revealed that decreased anoikis of Klotho-overexpressed Huh7 cells was also reversed by shRNA-mediated PAK1 knockdown (Figure 5E).

**Figure 6:**
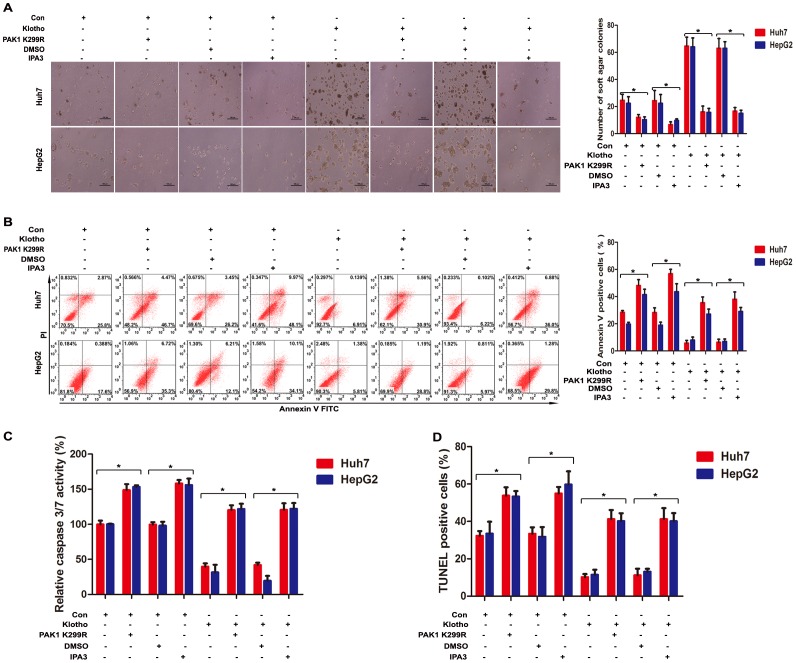
PAK1 kinase activity is required for Klotho-induced anoikis resistance. (A) Colony formation assay, (B) Annexin V/PI staining assay, (C) Caspase 3/7 activity assay, and (D) TUNEL assay after 48 hours detachment for Huh7 and HepG2 cells transfected with empty vector and Klotho plasmid, respectively, without or with PAK1 K299R cotransfection, and DMSO or IPA3 treatment. The graphic profiles represent the mean of three independent replicates in each group with standard error bars and were statistically analyzed with a *t* test (*, *P*<0.05).

To further elucidate the pivotal role of PAK1 kinase activity in Klotho-induced anoikis resistance, functional alterations were analyzed after PAK1 kinase activity inhibition with kinase dead mutant PAK1 K299R cotransfection and PAK1 allosteric inhibitor IPA3 administration in Klotho-overexpressed hepatoma cells. Colony formation assay showed that increased anchorage-independent growth of hepatoma cells due to Klotho expression was reversed by inactivating PAK1 kinase activity with PAK1 K299R cotransfection or IPA3 treatment (Figure 6A). Annexin V/PI staining (Figure 6B), Caspase 3/7 activity assay (Figure 6C), and TUNEL assay (Figure 6D) in suspended hepatoma cells showed that Klotho-induced anoikis resistance was reversed by inactivating PAK1 kinase activity with PAK1 K299R cotransfection or IPA3 treatment. These data demonstrate that Klotho-induced anoikis resistance is mediated by hyperactivation of PAK1.

### VEGFR2/PAK1 signaling arbitrates Klotho-induced resistance of hepatoma cells to anoikis

**Figure 7:**
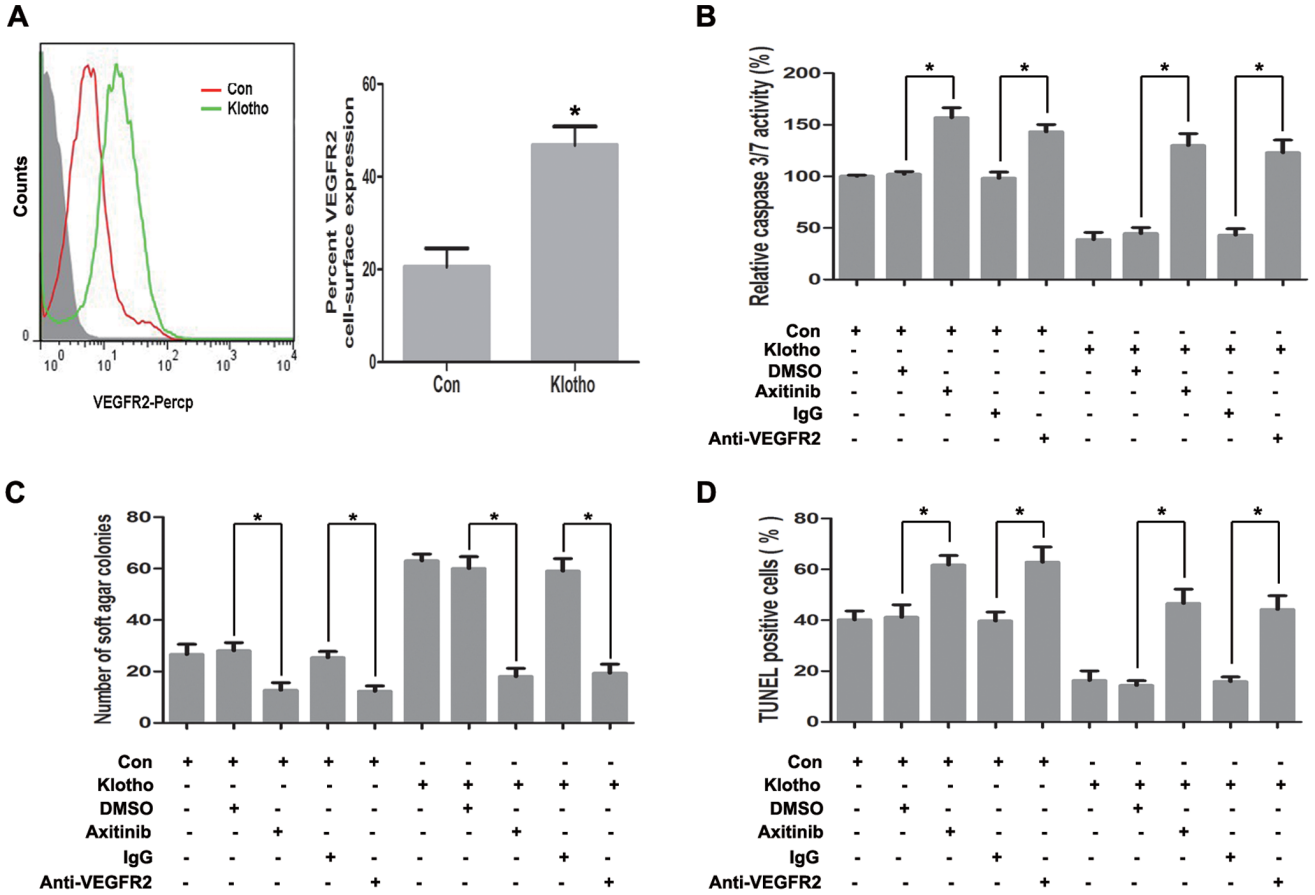
VEGFR2 elevation is involved in Klotho-induced anoikis resistance. (A) Flow cytometry analysis for VEGFR2 expression in huh7 cells transiently transfected with empty vector and Klotho plasmid, respectively. (B) Caspase 3/7 activity assay after detachment for 48 hours, (C) Colony formation assay, and (D) TUNEL assay after 48 hours detachment for Huh7 cells stably transfected with empty vector and Klotho plasmid, respectively, without or with DMSO or VEGFR2 inhibitor Axitinib, and isotype IgG or anti-VEGFR2 blocking antibody treatment. The graphic profiles represent the mean of three independent replicates in each group with standard error bars and were statistically analyzed with a *t* test (*, *P*<0.05).

Previous studies indicated that Klotho could associate with VEGFR2 to regulate Ca^2+^ influx [23], and Ca^2+^ influx is required for angiotensin II-induced activation of PAK1 [24]. Klotho expression was found to increase VEGFR2 protein level in hepatoma cells suggesting the potential function of VEGFR2 in Klotho-mediated anoikis resistance (Figure 7A). To illuminate the crucial function of VEGFR2 activation in Klotho-induced anoikis resistance, functional alterations were analyzed after VEGFR2 inhibition with VEGFR2 inhibitor Axitinib and its blocking antibody treatment in Klotho-overexpressed hepatoma cells. Caspase 3/7 activity assay (Figure 7B) and TUNEL assay (Figure 7D) in suspended hepatoma cells showed that Klotho-induced anoikis resistance was reversed by VEGFR2 inhibition with VEGFR2 inhibitor Axitinib or its blocking antibody. Colony formation assay showed that increased anchorage-independent growth of hepatoma cells due to Klotho expression were reversed by VEGFR2 inhibition with VEGFR2 inhibitor Axitinib or its blocking antibody (Figure 7C).

**Figure 8:**
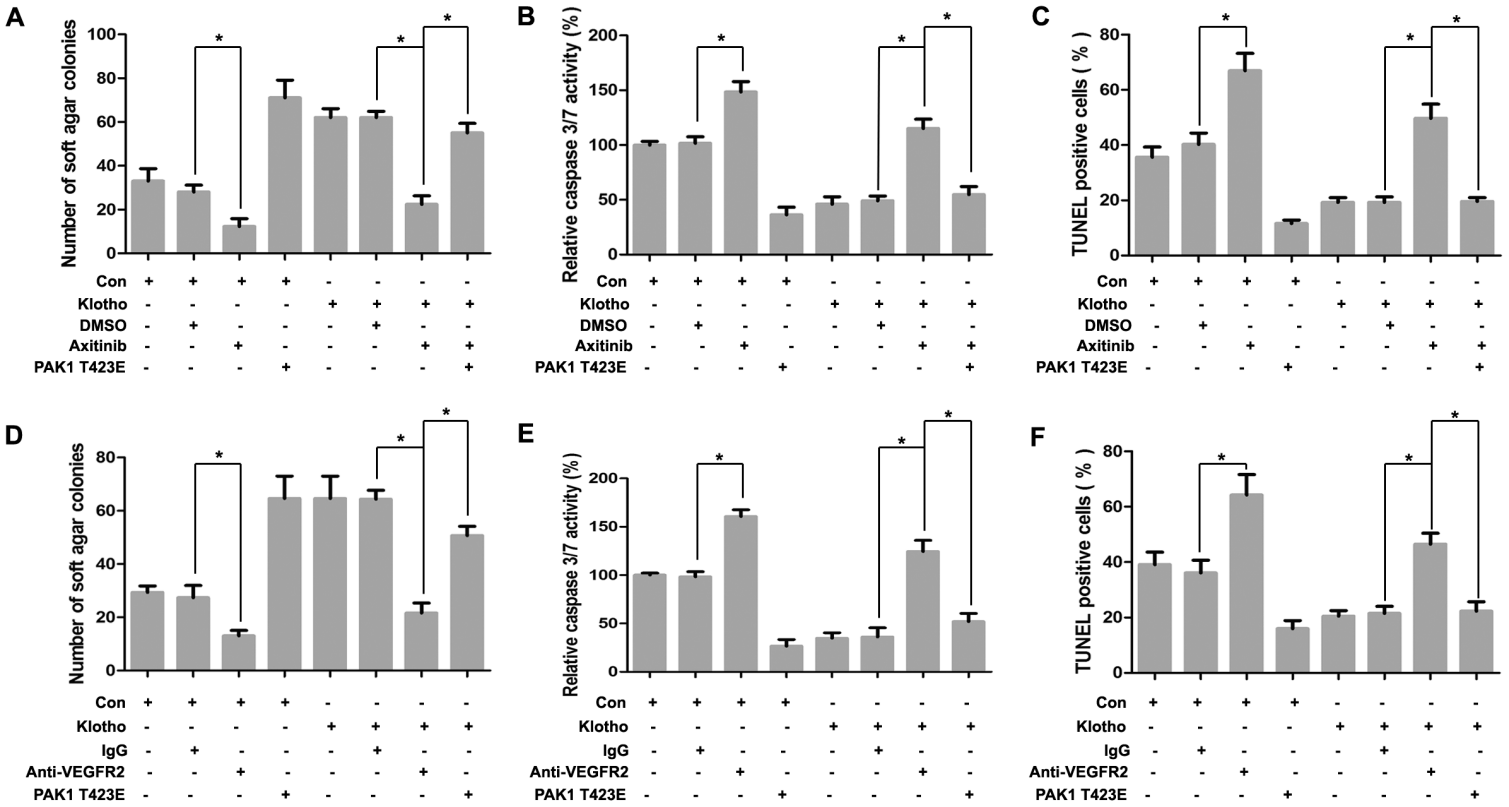
VEGFR2/PAK1 signal activation arbitrates Klotho-induced anoikis resistance. (A) Colony formation assay, (B) Caspase 3/7 activity assay and (C) TUNEL assay after 48 hours detachment for Huh7 cells stably transfected with empty vector and Klotho plasmid, respectively, without or with DMSO or VEGFR2 inhibitor Axitinib treatment in the absence or presence of constitutive active mutant PAK1 T423E cotransfection. (D) Colony formation assay, (E) Caspase 3/7 activity assay and (F) TUNEL assay after 48 hours detachment for Huh7 cells stably transfected with empty vector and Klotho plasmid, respectively, without or with isotype IgG or anti-VEGFR2 blocking antibody treatment in the absence or presence of constitutive active mutant PAK1 T423E cotransfection. The graphic profiles represent the mean of three independent replicates in each group with standard error bars and were statistically analyzed with a *t* test (*, *P*<0.05).

To further identify PAK1 activation as downstream effector of VEGFR2 signal in Klotho-mediated anoikis resistance, functional alterations were analyzed after PAK1 activation with constitutive active mutant PAK1 T423E cotransfection in the presence of VEGFR2 inhibition with VEGR2 inhibitor Axitinib or its blocking antibody treatment in Klotho-overexpressed hepatoma cells. Colony formation assay showed that inhibited anchorage-independent growth by VEGFR2 inhibitor Axitinib or its blocking antibody was partially reversed by PAK1 activation with PAK1 T423E cotransfection in Klotho-overexpressed hepatoma cells (Figure 8A, 8D). Caspase 3/7 activity assay revealed that increased anoikis by VEGFR2 inhibitor Axitinib or its blocking antibody was partially reversed by PAK1 activation with PAK1 T423E cotransfection in Klotho-overexpressed hepatoma cells (Figure 8B, 8E). TUNEL assay revealed that increased anoikis by VEGFR2 inhibitor Axitinib or its blocking antibody was partially blunted by PAK1 activation with PAK1 T423E cotransfection in Klotho-overexpressed hepatoma cells (Figure 8C, 8F).

**Figure 9:**
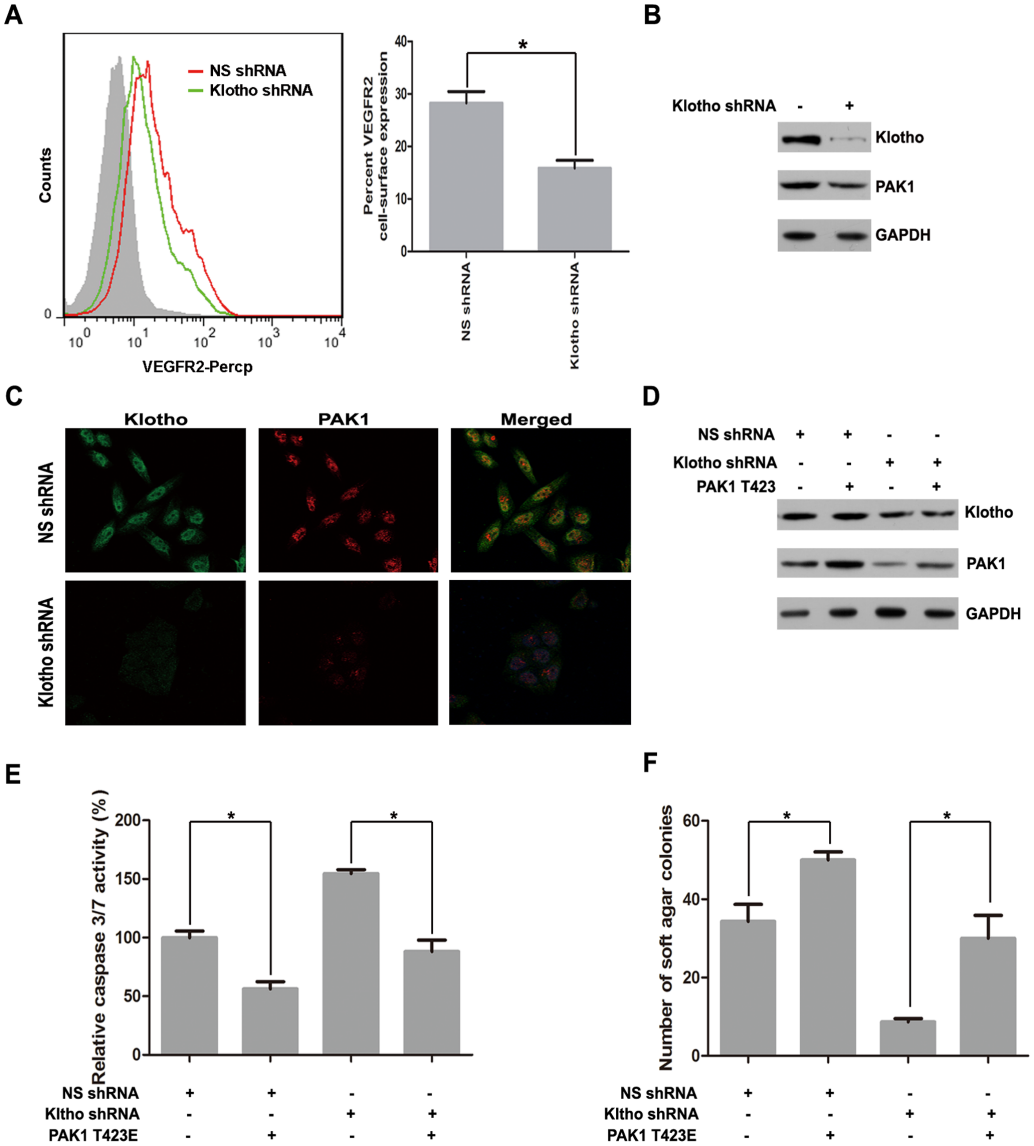
Decreased Klotho expression inhibits VEGFR2/PAK1 signaling. (A) Flow cytometry analysis for VEGFR2 expression in BEL7402 cells stably transfected with nonspecific shRNA, and Klotho 1# shRNA, respectively. (B) Western blot analysis of PAK1, Klotho and GAPDH protein levels and (C) immunofluorescence staining of PAK1 and Klotho for BEL7402 cells stably transfected with nonspecific shRNA, and Klotho 1# shRNA, respectively. (D) Western blot analysis of PAK1, Klotho and GAPDH for BEL7402 cells stably transfected with nonspecific shRNA, and Klotho 1# shRNA, respectively, in the absence or presence of constitutive active mutant PAK1 T423E transfection. (E) Caspase 3/7 activity assay and (F) Colony formation assay after 48 hours detachment for BEL7402 cells stably transfected with nonspecific shRNA and Klotho1# shRNA, respectively in the absence or presence of constitutive active mutant PAK1 T423E transfection. The graphic profiles represent the mean of three independent replicates in each group with standard error bars and were statistically analyzed with a *t* test (*, *P* <0.05).

To further validate the role of VEGFR2/PAK1 signaling in Klotho-mediated anoikis resistance, we investigated the VEGFR2 and PAK1 expression in Klotho-knockdown BEL7402 cells. Flow cytometry assay revealed that VEGFR2 expression was reduced in Klotho-knockdown BEL7402 cells (Figure 9A). Western blot (Figure 9B) and immunofluorescence staining (Figure 9C) revealed that PAK1 expression was decreased in Klotho-knockdown BEL7402 cells. Furthermore, reduced PAK1 expression by Klotho knockdown was partially reversed by PAK1 T423E cotransfection (Figure 9D). Caspase 3/7 activity assay (Figure 9E) revealed that increased anoikis by Klotho knockdown was partially reversed by PAK1 T423E cotransfection. Colony formation assay (Figure 9F) revealed that decreased anchorage-independent growth by Klotho knockdown was partially reversed by PAK1 T423E cotransfection. Immunohistochemical analysis showed that Klotho expression was positively correlated with PAK1 staining (n = 52, r = 0.5427, *P*<0.0001) in HCC tumor tissues (Figure S1). Taken together, these results suggest that VEGFR2/PAK1 signaling dominates Klotho-induced resistance of hepatoma cells to anoikis.

## Discussion

The present study demonstrates that Klotho expression is significantly associated with tumor aggressiveness and poor overall survival in a clinical follow-up of 52 hepatoma patients, indicating the potential oncogenic function of Klotho in hepatocarcinogenesis. We also provide evidence that Klotho expression confers hepatoma cells with resistance to anoikis via activating VEGFR2/PAK1 signaling, which could be reversed by inhibition with PAK1 allosteric inhibitor IPA3 or VEGFR2 inhibitor Axitinib administration in Klotho-overexpressed hepatoma cells. These findings identify Klotho/VEGFR2/PAK1 signal inhibition as a potential molecular targeting therapeutic intervention with anoikis resistance in advanced-stage HCC patients with metastasis.

As an essential senescence-suppressing factor, transmembrane and secreted forms of Klotho could exert pleiotropic activities in malignant transformation. The transmembrane form of Klotho functions as an obligatory co-receptor for fibroblast growth factor 23 (FGF23), a bone-derived hormone that regulates the phosphate, calcium and vitamin D balance, and defects in either Klotho or FGF23 cause phosphate retention and a premature-ageing syndrome in mice [25], [26]. The secreted form of Klotho regulates activities of multiple ion channels and growth factors including insulin, insulin-like growth factor-1 (IGF-1) and Wnt, and protects cells and tissues from oxidative stress through a mechanism yet to be identified [27], [28], [29], [30], [31], [32]. Secreted Klotho is found in the blood circulation acting as a peptide hormone. Studies have shown that secreted Klotho can suppress autophosphorylation of IGF type I receptor (IGF-IR) [29]. As IGFs are associated with the development of HCC [33], it is speculated that Klotho may be involved in hepatocarcinogenesis. However, some experiments show that secreted Klotho is able to stimulate angiogenesis and inhibit growth [30], [34], opposing its anti-IGF effect. Little is known about the serum level of Klotho in HCC and its association with tumor progression.

Klotho is identified as a potential tumor suppressor and an inhibitor of the IGF-1 pathway and activator of the FGF pathway in human breast cancer [35]. Klotho also inhibits transforming growth factor-β1 (TGF-β1) signaling and suppresses renal fibrosis and cancer metastasis in mice [36]. Klotho could also inhibit proliferation and increase apoptosis of human lung cancer A549 cells, which may be partly due to the inhibition of IGF-1/insulin pathways and involving regulating the expression of the apoptosis-related genes bax/bcl-2 [37]. However, Klotho expression is associated with epithelial ovarian cancer progression, and the protein may serve as an independent marker for ovarian cancer prognosis [38]. In contrast to previous studies indicating Klotho as a putative tumor suppressor gene in human breast cancer, kidney cancer and lung cancer, our present study reveals the oncogenic function of Klotho in hepatocarcinogenesis, which is consistent with previous findings in epithelial ovarian cancer. We provide evidence that immunohistochemical Klotho staining is associated with liver cancer progression and poor overall survival, indicating Klotho expression as a poor prognostic marker for survival in HCC patients who underwent surgical resection. These results may have important clinical implications for risk stratification and the planning of postsurgical surveillance for liver cancer patients.

Since anoikis resistance is prerequisite for hepatoma progression and metastasis, resistance of hepatoma cells to anoikis due to Klotho expression might be the crucial functional role of Klotho in HCC development and progression. Consistent with our previous studies indicating the pivotal significance of PAK1-induced anoikis resistance in HBV-associated hepatocarcinogenesis, our current data also indentify VEGFR2/PAK1 activation as downstream effector of Klotho expression in anoikis resistance of hepatoma cells. Besides, we have also provide evidence that VEGFR2/PAK1 inhibition with VEGFR2 inhibitor Axitinib, or anti-VEGFR2 blocking antibody, or PAK1 allosteric inhibitor IPA3 administration could significantly blunt anoikis resistance in Klotho-overexpressed hepatoma cells. These data suggest that Klotho/VEGFR2/PAK1 signal inhibition with VEGFR2 inhibitor Axitinib, or anti-VEGFR2 blocking antibody, or PAK1 allosteric inhibitor IPA3 administration might be used as potential therapeutic interventions with progression and metastasis of HCC patients, which awaits further clinical investigations. Although we have identified that Klotho expression confers hepatoma cells with resistance to anoikis through VEGFR2/PAK1 activation, the underlying molecular mechanism for Klotho-mediated VEGFR2/PAK1 activation remains still obscure and needs further exploration.

As a multikinase inhibitor targeting Raf, VEGFR and PDGFR signals, Sorafenib has been used as a first-line therapeutic drug and shown clinical efficiency for advanced-stage HCC patients [39]. Whether Sorafenib could exert its therapeutic function with anoikis sensitization or not in hepatoma cells merits further investigation. Given the crucial role of Klotho/VEGFR2/PAK1 activation in hepatoma resistance to anoikis in hepatocarcinogenesis, specific inhibitors directing VEGFR2 or PAK1 including Sorafenib, Axitinib and IPA3 might prove useful as anoikis-sensitizing therapeutic drugs for intervention with HCC metastasis. In our current study, VEGFR2 inhibitor Axitinib, anti-VEGFR2 blocking antibody, and PAK1 allosteric inhibitor IPA3 were identified as efficient inhibitors for Klotho-mediated anoikis resistance and anchorage-independent growth, which paves a potential avenue for personalized therapeutic intervention of hepatoma metastasis in HCC patients with high Klotho expression.

## Supporting Information

Figure S1 **Klotho expression positively correlates with PAK1 in tumor tissues from patients with HCC.** (A) Representative photomicrographs of immunohistochemical Klotho staining in human HCC tissues: Grade 0 for negative staining intensity, Grade I for weak staining intensity, Grade II for moderate staining intensity, and Grade III for strong staining intensity (original magnification, ×400). (B) A significant positive correlation between Klotho and PAK1 was shown (n = 52, r = 0.5427, P<0.0001).
(TIF)
