# Supplementary figures and images for: Klotho Endows Hepatoma Cells with Resistance to Anoikis via VEGFR2/PAK1 Activation in Hepatocellular Carcinoma
Source: PLoS One. 2013 Mar 13;8(3):e58413. doi: 10.1371/journal.pone.0058413 (PMC3596390; doi:10.1371/journal.pone.0058413)

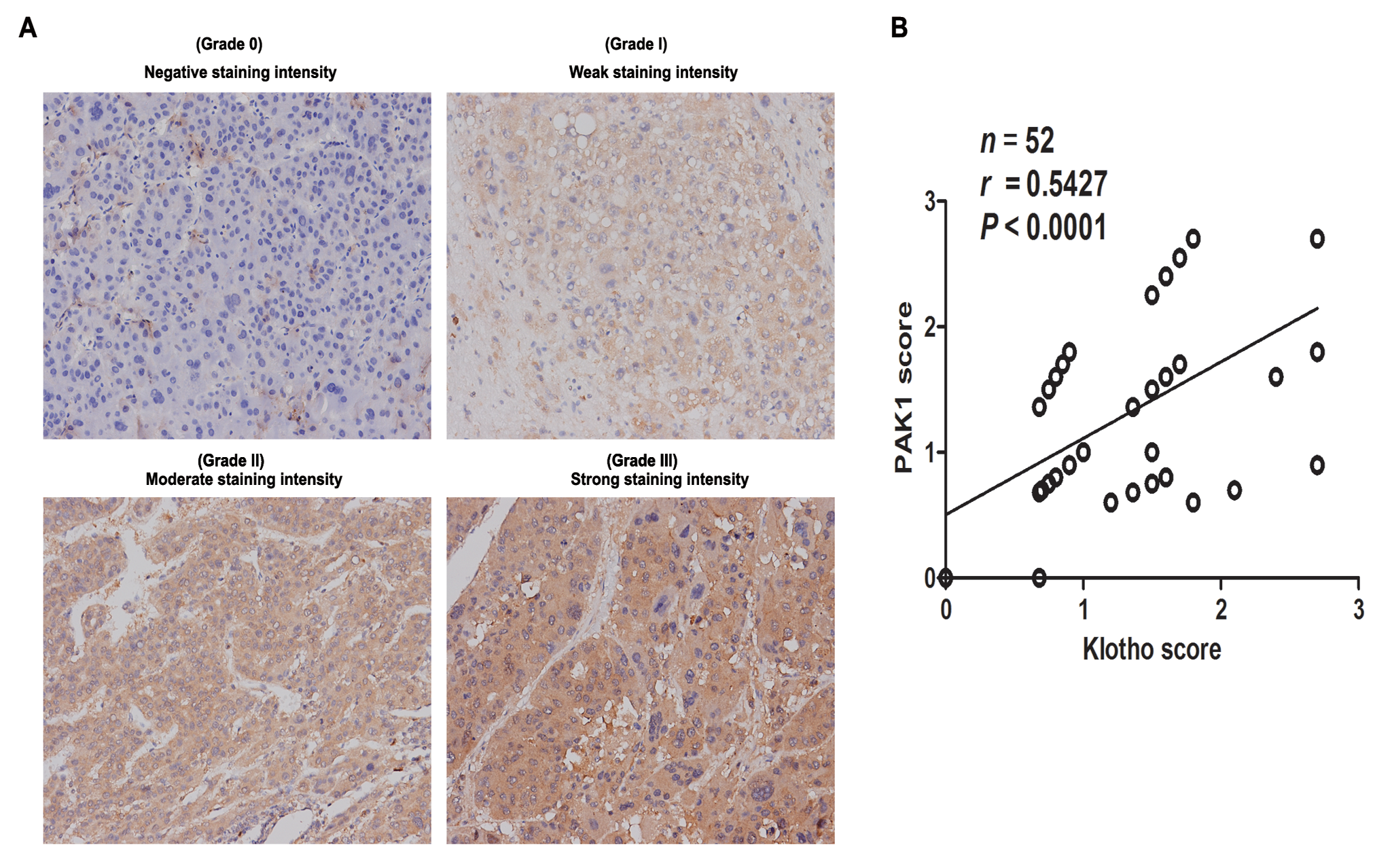

Supplement: Figure S1 — Klotho expression positively correlates with PAK1 in tumor tissues from patients with HCC. (A) Representative photomicrographs of immunohistochemical Klotho staining in human HCC tissues: Grade 0 for negative staining intensity, Grade I for weak staining intensity, Grade II for moderate staining intensity, and Grade III for strong staining intensity (original magnification, ×400). (B) A significant positive correlation between Klotho and PAK1 was shown (n = 52, r = 0.5427, P<0.0001). (TIF) [file pone.0058413.s001.tif]
